# Factors influencing the quality of preconception healthcare in China: applying a preconceptional instrument to assess healthcare needs

**DOI:** 10.1186/1471-2393-14-360

**Published:** 2014-11-03

**Authors:** Xinliang Zhao, Xiaoqing Jiang, Junzhen Zhu, Guozheng Li, Xiaoyan He, Fengying Ma, Qian Meng, Qinying Cao, Yucui Meng, Christopher Howson, Nanbert Zhong, Yaping Tian

**Affiliations:** Chinese PLA General Hospital/Medical School of Chinese PLA, Beijing, China; Peking University Center of Medical Genetics, Beijing, China; Jiangsu Provincial Center for Maternal and Children’s Health, Nanjing, Jiangsu Province China; Hebei Provincial People’s Hospital, Shijiazhuang, Hebei Province China; The No. 4 Hospital of Shijiazhuang City, Shijiazhuang, Hebei Province China; Hebei Institute of Reproductive Science and Technology, Shijiazhuang, Hebei Province China; Yangzhou Municipal Maternal and Children’s Hospital, Yangzhou, Jiangsu Province China; Wuxi Municipal Maternal and Children’s Hospital, Wuxi, Jiangsu Province China; Lianyungang Municipal Maternal and Children’s Hospital, Lianyungang, Jiangsu Province China; March of Dimes Global Network for Maternal and Infant’s Health, White Plains, NY USA; China Foundation of Birth Defect Intervention and Aid, Beijing, China; Children’s Hospital of Shanghai, Jiaotong University, Shanghai, China; New York State Institute for Basic Research in Developmental Disabilities, Staten Island, NY USA

**Keywords:** Preconception healthcare, Maternal health, Survey, China

## Abstract

**Background:**

Preconception care is defined as the promotion of the health and well-being of a woman and her partner before pregnancy. Improving preconception health can result in improved reproductive health outcomes. China has issued latest version official guideline for preconception care in 2011. The objective of this cross-sectional study is to determine whether there is a variation in the quality of preconception healthcare services in distinct eastern and northern populations of China, and what factors are associated with such variation.

**Methods:**

A cross-sectional survey using our previously developed preconception instrument was conducted. Women at reproductive age planning for pregnancy were surveyed along with their partners at hospitals during their pre-pregnancy health examination. Data collected include general health/life profiles, pregnancy history, alcohol/tobacco/drug exposures, immunizations, micronutrient supplements and the demands in preconception care. After quality assessment, statistical analysis were applied to evaluate the variations in preconception factors between people from Hebei and Jiangsu Provinces.

**Results:**

3202 women of reproductive age in from eastern province, Jiangsu, and in a northern province, Hebei, participated this study. 2806 of them and their partners have completed the questionnaire, at a rate of 87.6%, 1011 were from Jiangsu and 1795 were from Hebei. Statistical significance was obtained for maternal age (P < 0.001), body mass index (u =13.590, P <0.001), education (χ^2^ = 916.33, P < 0.001), occupation (χ^2^ = 901.78, P < 0.001), health status/common disease, immunization status, and need for preconception care.

**Conclusions:**

For a country as large as China, the centralized guideline for standardized preconception healthcare does have a very crucial positive role in reproductive healthcare, but it may not be suited for all populations. Regional authorities should consider the demographics and healthcare needs of the local population and modify the centralized guideline accordingly, as well as provide a better education and professional services for the public, to improve the quality of preconception services at both the regional and the national level.

## Background

Preconception care is defined as the promotion of the health and well-being of a woman and her partner before pregnancy [1]. Improving preconception health can result in improved reproductive health outcomes, with the potential for reducing societal costs as well [2]. Preconception care aims to promote the health of women of reproductive age before conception and thereby improve pregnancy-related outcomes [3]. Screening couples who intend to conceive should provide accurate information about their health state and cover a broad range of topics, including general lifestyle, nutrition, tobacco and alcohol use, obstetric and medical history, and genetic conditions in either parent or family.

Since 1987, several reviews have analyzed the evidence from, and documented the effectiveness of, specific preconception interventions [2, 3]. The systematic review in 2002 by Korenbrot *et al*. of 21 research trials published during the 1990s strengthened the evidence base for preconception care in particular areas [4]. The United States Centers for Disease Control and Prevention published recommendations to improve preconception health and health care in 2006 [4]. Afterwards, Netherlands and Canada issued their own national recommendations or guidelines for preconception care [5, 6]. 2012 World Health Organization issued a report and packages to strengthen the preconception care in order to reduce maternal and childhood mortality and morbidity [7].

Pre-marriage and preconception healthcare services have been provided by the Chinese government since 1980, and a nationwide maternal healthcare network is well established. The preconceptional healthcare service package has been upgraded many times. An official guideline for preconception healthcare was developed many years ago, and its latest version was released in 2011 by the Chinese Medical Association [8]. The importance of preconception healthcare has long been established, and various studies have emphasized its benefits in China [9–11]. However, detailed assessment of the needs for preconception health care, especially when comparing economically developed south China to still-developing north China, has seldom been conducted. As a large country with the highest population in the world, China has numerous geographical features widely distributed throughout the territory, and its economic statuses and life styles vary between regions, as do the general health indicators [8, 12]; the country is also experiencing shortages and unbalanced distribution of health care services [13]. Therefore, the current maternal health care system must be assessed to maximize the effect of preconception services, understand the basic needs of couples, and design a strategy that best fits the preconceptional needs.

We conducted this study to evaluate the variation of risk factors for adverse pregnancy outcome, intentions, and needs for preconception services between the eastern and northern regions in China and to discuss the best strategy for strengthening preconceptional health care to meet the increased needs.

## Methods

### Participants

Two distant provinces, Jiangsu and Hebei, were selected because of the differences in their environmental, geographical, and economical features and in their residents’ lifestyles. Jiangsu province is in the plains region of east China and is known by its characteristic urban lifestyle and its middle-class to wealthy living standard, whereas Hebei is a mountainous province located in north China and is known for less developed rural life and lower standard of living; both provinces are coastal. This project has been reviewed and approved by the Institutional Ethics Committee in both Jiangsu Provincial Center for Maternal and Children’s Health and Hebei Provincial People’s Hospital.

A questionnaire we developed earlier as a preconceptional instrument was based on the common risk factors for pregnancy complications and couples’ needs. The survey was conducted in Jiangsu and Hebei provinces independently. Three geographically separated hospitals were selected as the data collection fields for each province. The survey period was from March 2010 to May 2012.

Couples who intended to conceive were recommended to hospital for pre-pregnancy health examination, which is provided by the government for free of charge. Any couples applied for pre-pregnancy health examination were asked to participate this study. Once obtained agreement, our trained staff will facilitate them to fill in the questionnaire. The hard copy of questionnaires was double-entered into Excel (Microsoft) spreadsheets, after which an audit was performed for quality assessment to rule out all unqualified, incomplete, or incorrect entries. Data were analyzed using SPSS version 17.0 for Windows (Chicago. IL, USA). Pearson Chi-square test was used to test qualitative associations if all expected frequencies are equal to or greater than 5, Fisher’s test would be used when the expected frequencies are less than 5. Mann–Whitney test was used for testing difference between two sets of continuous variables, when with equal variances; and Wilcoxon Rank Sum test was used in heterocedasticity. A P*-*value of 0.05 or less was used to define statistical significant results.

## Results

We have introduced the questionnaires to 3946 couples and 3,202 pairs agreed to participate, with the response rate of 81.1%. Among them, 1,173 were from Jiangsu and 2,029 were from Hebei, respectively. Then the incomplete or unqualified questionnaires were ruled out, 2,806 questionnaires (1,011 from Jiangsu and 1,795 from Hebei) were determined to be valid for further analysis.

### Women’s pregnancy history and outcomes

Details of women’s pregnancy history and outcomes were shown in Table 1. Women are classified as primiparous or multiparous. Although China’s “One-child policy” started in 1979, many provinces have updated the policy that allows a couple to have two children if both parents have no sibling after 2005. In Jiangsu province, 435 (43.0%) participants had a previous history of pregnancy. Of these participants, 287 (28.4%) had had an abortion (induced); 132 (13.1%) had had a miscarriage (spontaneous); 8 (1.8%) had had a stillbirth; and 126 (29.0%) had had a successful delivery, giving birth to 128 infants. Of these 128 infants, 9 were premature, 5 were low in birth weight, and 14 were reported to be affected by birth defects. In Hebei province, 1,004 (55.9%) participants had a previous history of pregnancy. Of these participants, 273 (15.2%) had had an abortion; 87 (4.8%) had had a miscarriage, 19.4% of which occurred after abortion (medically induced); 28 (10.3%) had had a still birth; and 826 (82.3%) had successful deliveries, giving birth to 829 infants. Among these infants, 17 (2.1%) were premature, 15 (1.8%) were low in birth weight, and 16 (1.9%) were reported to be affected by birth defects.

**Table 1:** **General life indicators and pregnancy history of the female participants**

	Jiangsu (n = 1011)		Hebei (n = 1795)		Comparison
	N (%) or median (IQR)				
Age, y	28.3 (IQR = 4)		27.3 (IQR = 6)		P <0.001 (Rank Sum)
15–18	0	—	1	(0.1%)	
18–25	239	(23.6%)	673	(37.5%)	
26–35	733	(72.5%)	1007	(56.1%)	
36–45	39	(3.9%)	112	(6.2%)	
45+	0	—	2	(0.1%)	
Occupation					χ^2^ = 901.78, P < 0.001
Unemployed	266	(26.3%)	1140	(63.5%)	
IT	2	(0.2%)	10	(0.6%)	
Sales	43	(4.3%)	30	(1.7%)	
Office	386	(38.2%)	65	(3.6%)	
Service	94	(9.3%)	210	(11.7%)	
Labor	87	(8.6%)	125	(7.0%)	
Medical	34	(3.4%)	9	(0.5%)	
Teaching	93	(9.2%)	34	(1.9%)	
Student	2	(0.2%)	0	—	
Legal	4	(0.4%)	0	—	
Peasant	0		172	(9.6%)	
Education					χ^2^ = 916.33, P < 0.001
Illiteracy	10	(1.0%)	4	(0.2%)	
Below Primary	7	(0.7%)	8	(0.4%)	
Primary	29	(2.9%)	74	(4.1%)	
High school	318	(31.5%)	1522	(84.8%)	
University & above	647	(64.0%)	187	(10.4%)	
Place of living					χ^2^ = 1583.2, P < 0.001
Urban	806	(79.7%)	113	(6.7%)	
Rural	205	(20.3%)	1682	(93.7%)	
Number of people living with					χ^2^ = 110.46, P < 0.001
1	469	(46.3%)	659	(36.7%)	
2	166	(16.4%)	275	(15.3%)	
3	257	(25.4%)	346	(19.3%)	
4	93	(9.2%)	433	(24.1%)	
5 and more	26	(2.6%)	82	(4.5%)	
Number of rooms per person in the house/apartment					χ^2^ = 23.17, P < 0.001
1 and less	201	(19.9%)	425	(23.7%)	
2	615	(60.8%)	1140	(63.5%)	
3 and more	195	(19.3%)	229	(12.8%)	
Previous pregnancy					
History of pregnancy	435	(43.0%)	1004	(55.9%)	χ^2^ = 16.19, P = 0.001
History of Abortion	287	(28.4%)	273	(15.2%)	χ^2^ = 70.32, P < 0.001
History of miscarriage	132	(13.1%)	87	(4.8%)	χ^2^ = 4.3, P = 0.038
History of stillbirth	8	(1.8%)	28	(10.3%)	χ^2^ = 3.02, P = 0.082
History of successful delivery	126	(29.0%)	826	(82.3%)	χ^2^ = 324.82, P < 0.001
Infants produced	128		829		
Premature	9	(7.0%)	17	(2.1%)	P < 0.004 (Two-tailed)
Low birth weight	5	(3.9%)	15	(1.8%)	P = 0.171 (Two-tailed)
Birth defects	14	(10.9%)	16	(1.9%)	P < 0.001 (Two-tailed)

Abortion: induced abortion.

Miscarriage: spontaneous miscarriage.

Statistical significance were identified for the variation in the history of pregnancy, abortion, miscarriage, successful delivery, and also for prematurity and birth defects between two provinces.

### General health indicators

The details of the general life indicators of female participants are shown in Table 1. The age of female participants were compared with rank sum test. The results show statistical significance (P <0.001). In Jiangsu province, the female participants’ body mass index (BMI) ranged from 15.62 to 38.90, and the average BMI was 21.01. Most (79.53%, 804/1011) of these females’ BMIs were in the normal range, whereas 14.34% (145/1011) were underweight, 5.74% (58/1011) were overweight (obesity excluded), and 0.4% (4/1011) were obese (overweight excluded). In Hebei province, the female participants’ BMI ranged from 11.67 to 37.78, and the average BMI was 22.15. Most (77.83%, 1397/1795) of these females’ BMIs were in the normal range, 7.52% (135/1795) were underweight, 13.04% (234/1795) were overweight (obesity excluded), and 1.62% (29/1795) were obese (overweight excluded) [14]. The differences in BMI between the two provinces’ participants were assessed with Mann–Whitney test, and the results showed significance (u = 13.590, P < 0.001).

In Jiangsu province, the height of female participants varied from 140 cm to 178 cm, and the average height is 162 cm. In Hebei province, the height of female participants varied from 137 cm to 178 cm, the average height is 161 cm, and the median height is 160 cm. The differences in height between the two provinces’ participants were assessed with Mann–Whitney U test and showed significance (u = 13.590, P < 0.001).

In Jiangsu province, the weights of female participants varied from 40 kg to 97 kg, with a mean weight of 54.85 kg. In Hebei province, the weights of female participants varied from 31 kg to 95 kg, with a mean weight of 57.31 kg. Because the weight of the participants were not normally distributed, the Mann–Whitney U test was performed to analyze the differences between the weights for the two provinces. The results show significance (u = 13.590, P < 0.001).

### Life related issues

Relative data were shown in Table 1. Among the participants collected in this study, over 50% of the participants in Jiangsu have a college degree, but 84.8% of the participants from Hebei are educated only to the high school level. 38.2% of Jiangsu’s participants are office workers, whereas 63.5% of Hebei’s participants are unemployed.

Living environment of the participants differs. In Jiangsu, most of the participants (79.72%, 806/1011) live in urban or suburban areas, whereas in Hebei, most participants (93.70%, 1682/1795) live in rural areas. Less than 50% of the participants from both provinces are living with their partners alone, for less disturbance from senior relatives. Interestingly, 28.6% of the participants from Hebei live with four or more relatives. Despite the quality of the rooms in their homes, approximately 80% of the participants from both provinces have more than one room in their homes per family member.

The questionnaire also covered alcohol, tobacco and illicit drug exposures of women in the past 90 days. Only 7.8% of Jiangsu participants, and 2.3% of Hebei participants have taken alcohols in the form of either spirit, wine or beer. Tobacco smoking is less common in the provinces, with 1.2% and 0.4% for Jiangsu and Hebei, respectively. The use of illicit drugs is extremely rare: only one case was reported in Jiangsu, and none in Hebei.

### Common infectious diseases and chronic diseases

Chronic diseases (hypertension, anemia, diabetes, periodontitis, hypothyroidism, and heart failure), infectious diseases (sexually transmitted disease, STDs; intestinal parasitic infection), and mental status (history of depression, heavy stress) were assessed in the survey. Anemia and periodontitis were each found in more than 5% of Jiangsu’s participants. In contrast, these diseases were found in 1.03% and 0.25% of Hebei participants, respectively. In addition, 1.53% and 0.25% of Jiangsu’s and Hebei participants, respectively, reported that they experience heavy stress in everyday life.

### Immunization and supplement intake

Details of the women’s immunization and supplement intake were shown in Table 2. Three common immunizations were included in the survey: measles, mumps, and rubella (MMR); tetanus; and hepatitis B virus (HBV). The numbers of participants who received all three vaccinations are much higher in Jiangsu than in Hebei.

**Table 2:** **Immunization and micronutrient supplement of the female participants**

	Jiangsu		Hebei		Comparison
	N(%)		N(%)		Chi-square
Immunized					
MMR	894	(88.4%)	264	(14.7%)	χ^2^ = 1450.13, P <0.01
Tetanus	341	(33.7%)	92	(5.1%)	χ^2^ = 405.47, P <0.01
HBV	616	(60.9%)	585	(32.6%)	χ^2^ = 212.16, P <0.01
Taking micronutrient supplement					
Folate	424	(41.9%)	783	(43.6%)	χ^2^ = 0.75, P >0.05
Iron	26	(2.6%)	103	(5.7%)	χ^2^ = 14.78, P <0.01
Calcium	71	(7.0%)	202	(11.3%)	χ^2^ = 13.18, P <0.01
Multi-vitamin	124	(12.3%)	44	(2.5%)	χ^2^ = 110.66, P <0.01
iodized salt	815	(80.6%)	1153	(64.2%)	χ^2^ = 82.84, P <0.01

MMR: measles, mumps and rubella.

HBV: hepatitis B virus.

### Further needs in preconception health care service

Table 3 lists the issues in preconception health care which are concerned and required by the couples participated the study. The issues selected most frequently by participants from Jiangsu’s were folate and iodized salt supplementation, education on pregnancy health check-ups, micronutrient supplementation, environmental risks, birth spacing, STD prevention, and healthy weight maintenance. The topics selected most frequently by Hebei’s participants were education on pregnancy health check-ups, micronutrient supplementation, environmental risks, and birth spacing. Less than 20% of the participants from both provinces required improvements in immunization and contraception, and 2.3% of Jiangsu participants and 4.8% of Hebei participants selected MMR vaccination as an additional requirement in preconception. A major variation in the needs for education on genetic risks was identified between the participants from two provinces. This topic was selected by 23.9% of Jiangsu’s couples and 57% Hebei’s couples.

**Table 3:** **The couples’ needs to improve preconception health care**

	Jiangsu		Hebei		Rate ratio	95%CI
	N(%)		N(%)			
Immunization						
MMR	23	(2.3%)	86	(4.8%)	0.479	0.301-0.747
Tetanus	24	(2.4%)	36	(2.0%)	1.200	0.710-1.973
HBV	123	(12.2%)	326	(18.2%)	0.670	0.553-0.812
Supplements						
Folate	805	(79.6%)	943	(52.5%)	1.516	1.436-1.600
Iron	191	(18.9%)	500	(27.9%)	0.678	0.585-0.786
Calcium	221	(21.9%)	600	(33.4%)	0.656	0.572-0.747
Iodized salt	535	(52.9%)	799	(44.5%)	1.189	1.100-1.285
Knowledge						
Birth spacing	725	(71.7%)	1360	(75.8%)	0.947	0.903-0.992
STD prevention	551	(54.5%)	1043	(58.1%)	0.938	0.875-1.005
Genetic risk	242	(23.9%)	1024	(57.0%)	0.420	0.373-0.472
Healthy weight maintenance	688	(68.1%)	1271	(70.8%)	0.961	0.913-1.012
Micronutrient supplement	792	(78.3%)	1405	(78.3%)	1.001	0.961-1.042
Environmental risk	720	(71.2%)	1392	(77.5%)	0.918	0.877-0.962
Health check-up during pregnancy, when, why, how	825	(81.6%)	1407	(78.4%)	1.044	1.004-1.084
Other needs						
Contraception	338	(33.4%)	303	(16.9%)	1.981	1.731-2.266

## Discussion

The surveyed populations were carefully selected for the provinces’ characteristic geographical and socioeconomic statuses. Jiangsu province, which is in the Yangtze River Delta Economic Zone neighboring to Shanghai, is the modern region with a well-developed socioeconomic environment that may represent all of the provinces on the east coast of China. Hebei province, a northern region surrounding Beijing but covered in its western area by the Taihang Mountains, most of the regions are still under-developed. The ethnic compositions of the two provinces are very similar, with Han people (majority of the Chinese) weighted over 95% [15]. The climates of the two provinces are distinct, as the annual average temperature of Jiangsu is 2°C higher than that of Hebei, and Jiangsu has an annual rain fall of 704–1250 mm, which is nearly double that of Hebei [16, 17].

### General health indicators

In the statistical analysis of the results, many differences in general health indicators and lifestyle were identified between the two groups of participants. Women older than 35 years of age were classified as advanced maternal age and were informed that a longer period may be required to achieve conception and that there would be increased risks of pregnancy complications and chromosomal aberrations for the fetuses. These women were also informed about effective screening methods for the detection of fetal chromosomal aberrations [18]. The participants from Jiangsu were generally older in age than those from Hebei. Considering the occupational construction of two groups of participants, this could conclude that the average reproductive age of the urban population is one year higher than that of the rural population. Participants in both provinces specified the need for enhanced preconception education for healthy maternal age, and for pregnancy management of women of advanced maternal age, especially in Jiangsu province, which has a greater proportion of women with advanced maternal age.

BMI is an officially accepted tool to evaluate body fat, and it directly influences the pregnancy. A large-scale epidemiological study reported that the overweight (obesity included) rate is higher for the northern China population than for the southern China population [19]. Increased BMI is associated with an increased risk for infertility [20], gestational diabetes, and hypertension [21]. In Hebei, the proportions of the overweight and the obese are higher than in Jiangsu. A higher rate of underweight for Jiangsu participants has been noted. Underweight has been shown to be associated with an increased risk of preterm deliveries, low birth weight, and anemia [22]. Both extremes of maternal BMI showed a strong association with pregnancy complication and perinatal outcomes. Awareness must be raised, and more education and actual activities should be provided to reproductive-age women, addressing healthy weight management for both the underweight and the obese, specifically and respectively.

Education level and occupation reflect individuals’ level of knowledge, ability to understand information, and earning power, thus may also link to their economic status. Higher proportions of university graduates with master and doctoral degree and white collar workers were found in Jiangsu’s participants. People with more education can understand preconception services better and have greater concern and a more positive attitude about health-related issues [23]. Studies have shown that more highly educated people have greater intention to prepare for/manage their pregnancy scientifically [18, 24, 25]. The lower education level of rural women greatly limits their intention to access preconception service, resulting in less involvement in preconception health care [18]. A study of low-income Mexican Americans suggested that among women of child-bearing age, younger ages were associated with less knowledge [26]. The lower average age of the participants from Hebei may correlate with their lower education level. Improving education level and raising awareness are needed. Instead of treating all patients in a standard, routine fashion, doctors should provide clearer and more detailed explanations for less educated populations.

### Status of diseases that may affect pregnancy

Infectious disease can impact pregnancy-related outcomes and the reproductive health of women. Preconception screening for STDs, including HBV and human immunodeficiency virus (HIV), and hypothyroidism is provided by the Chinese government in the preconception health care package [8]. A benefit of preconception screening for STDs with appropriate treatment was the reduced rate of preterm birth [27]. The prevalence of infectious and most chronic diseases is below 0.5% for both provinces. The prevalence of anemia and periodontitis both were over 5% in Jiangsu, and lower in Hebei. Untreated anemia before pregnancy may lead to preterm labor, low birth weight [28], fetal growth retardation, and infantile developmental delay [29], and if postpartum hemorrhage occurs, it may cause death. Periodontitis mainly affects the delivery time and elevates the risk of preterm labor [30]. Researches on anemia and periodontitis carried out by other Chinese groups have shown that the prevalence for both diseases is higher in rural regions than in urban areas [31, 32]. Our finding in current study illustrated the opposite finding, that the prevalence for both diseases were lower in less developed Hebei Province. Together with the abnormally low prevalence of anemia and periodontitis in this study, the only possible explanation could be that the questionnaires were not filled in by medical professionals with screening instruments in hands but by the couples, and many mild cases were ignored [33]. Thus more preconception educational programs are required to improve the awareness and knowledge on the adverse effects of these common chronic diseases.

A stressful psychological state may lead to miscarriage, preterm delivery, low birth weight, or fetal brain developmental disorders as well as the risk of the baby developing schizophrenia and related disorders in later life [34–36]. During preconception counseling, once a woman is identified with a history of depression, severe psychological stress, or anxiety, she will be referred for psychological counseling to reduce the level of stress, if the facility is available locally. The occurrence of psychological problems among Jiangsu’s participants is higher. Some researchers reported that the psychological burden for the urban population is greater than for the rural population [37], and its occurrence increased with higher education level groups [38]. The leading causes of such burden in reproductive-age women are work and family-related issues [39]. Therefore more attention should be drawn to the psychological problems of the urban population not only for preconceptional health but also for normal life quality.

### Life style

For both provinces the alcohol consumption were rarely occurred among female participants, much lower than the data reported by Current Status of Alcohol consumption in China, which found 18.3% women drunk alcohol more than once in three months [40]. Fetal alcohol syndrome (FAS) is more likely to occur after continuous or heavy intake of alcohol during pregnancy. Effects have also been observed after intermittent or binge drinking during pregnancy [41, 42] and even from lower-level alcohol use. Other factors such as timing of exposure, maternal or fetal genetic factors affecting metabolism, or individual susceptibility coupled with other harmful behaviors can all contribute to determining the outcome of an alcohol-exposed pregnancy. The lower limit of alcohol intake at which no adverse effect will occur for any developing fetus has not yet been determined and may not even exist [42, 43]. Thanks for the vast preconceptional education campaigns for avoiding unhealthy life style before pregnancy, most Chinese women avoid alcohol consumption during pregnancy by their own intention [44], but for certain minor ethnic groups, the potential for alcohol consumption during pregnancy is higher, due to their unique habit and religion [45].

Apart from increased risks of cancer and pulmonary and cardiovascular disease, smoking can also lead to premature birth, sudden infant death, and ectopic pregnancy, and it has implications for the umbilical cord during pregnancy [46]. Since 1987, lung cancer has outpaced breast cancer as the leading cause of cancer death among women in the world, and exposure to environmental tobacco smoke (ETS) is a cause of lung cancer and coronary heart disease among lifelong nonsmokers. Moreover, infants born to women who are exposed to ETS during pregnancy may also have increased health risks, including small decreases in birth weight and slightly increased risk for intrauterine growth retardation, compared with infants born to women who were not exposed. In the U.S., 20% of women smoked cigarettes in 2003 [47], and 11.4% of women giving birth reported smoking during their pregnancy [48]. In China, 2.4% of women smoke, mostly between the ages of 20 and 39 years [49]. In comparison, the tobacco smoke rate were both lower than national average, especially in Hebei province. The preconceptional education campaign is also major driving force for the reduction among these child-bearing age participants.

Alcohol consumption, smoking, and drug consumption have been maintained at a low level from the preconception period; the current strategy appears to be effective in preventing unhealthy life styles that are detrimental to pregnancy health.

### Micronutrient supplementation

The use of five common micronutrients—folate, iron, calcium, multi-vitamins, and iodized salt—was assessed in the survey. All of these micronutrients are beneficial for pregnancy: folate and iodized food can reduce the risk of neural tube defects and hypothyroidism, and iron and calcium are effective for preventing pregnancy anemia and pregnancy osteoporosis. All the supplements are commercially available in both provinces. Apart from folate, the status of supplement intake of iron, calcium, multi-vitamin, and iodized salt in the two groups of participants differed. Hebei participants have more intention to take iron and calcium, whereas Jiangsu participants prefer multi-vitamins and iodized salt. In both provinces, less than 50% of the women took folic acid, although the supplement was provided free of charge by local maternal health clinics. Other studies reported similar findings of low intake rate of the free folic acid [45, 50]. The causes of low usage of folic acid are thought to be insufficient knowledge of the effect of micronutrients on healthy pregnancy, partners’ attitude, and doctors’ guidance, deduced by Liu *et al.* [50]. Thus, proper education should be provided to both women and their partners to improve the understanding of the important of micronutrient supplementation during preparation for conception and within the gestation, at the same time the service standard of doctors also needs to be ensured.

### Immunization

In this study we monitored immunizations for HBV, Tetanus and MMR. The overall immunization rate for Jiangsu’s female participants was greatly higher than the Hebei’s. Many vaccine-preventable diseases may have serious consequences for both mother and fetus during pregnancy, which makes the immunization status of women of reproductive age an important focal point for preconception care. The prevalence of HBV infection is high. According to a national survey of China published in 2008, 93 million people were infected by HBV, of which 30 million are patients affected with hepatitis B [51]. There is a 10–90% possibility of neonatal transmission of HBV [52]. In addition, infants who are exposed to acute infection *in utero* have a risk of low birth weight and prematurity [53]. Prevention of congenital rubella syndrome is a prototype for preconception care because the vaccination is needed before conception and is very effective in preventing a congenital disease that has significant morbidity and mortality rates. The prevention of rubella normally comes as part of the MMR vaccine, which has been found to be very efficacious for all three viral illnesses [54]. Tetanus is a condition that is caused by the inoculation of *Clostridium tetani* spores, which are widely distributed throughout the environment. Neonatal infection is extremely rare. There is no evidence that the tetanus toxoids vaccine is teratogenic when used extensively. The rate of immunization with the HBV, MMR, and tetanus vaccines is much higher in Jiangsu than in Hebei. A study of HBV immunization suggested that the immunization rate strongly correlated with awareness [55]; other studies also suggested that familial economic status and level of education also affect the rate of immunization [56]. Therefore, apart from the preconception education for immunization is urgently demanded in Hebei, enhanced guidance system operated by medical professionals is also required.

### Further needs reported by participants

The existing preconception health care network is under the administration of the National Committee of Family Planning and was originally established for the implementation of the “One-child policy”. Contraception assistance used to be its first priority. Preconception health care was introduced to the system after 2000, and the national general guideline for providing preconception health care was issued in 2007 and then renewed in 2011 [8]. Folic acid supplementation, education on environmental and genetic risks to pregnancy, and healthy weight maintenance are included in the guideline. However, the education doctors provide on these topics is usually routine and impersonal, lasting only 10 minutes, if that long—a booklet often substitutes for the whole education, due primarily to the shortage of preconception doctors.

Other topics such as immunization and micronutrient supplementation are generally absent from the preconception education. Patients frequently asked questions about micronutrient supplementation before and during pregnancy, and the doctors’ answers varied largely, as no official document was issued for this topic. After the “Population and family planning regulations” changed from 2003 to 2005, and allowed a second child if both sides of the couple have no sibling, questions about birth spacing were asked with increasing frequency. In current study, more than 70% of the participants from both provinces requested information about birth spacing. Inclusion of information on STD prevention is not mandatory in the service; however, some sites that have set up such education have seen encouraging results [57].

Although vaccination in the preconception period has been shown to have beneficial effects, immunization is excluded from the guideline [58]. No evidence was found that preconception immunization has been implemented on a large scale in China, although suggestions to be immunized before becoming pregnant can be found both on the Internet and printed media released by the Ministry of Health. The U.S. Centers for Disease Control and Prevention issued a list of vaccinations that are recommended in the preconception period, which includes MMR, Tetanus, HBV, influenza, varicella, diphtheria, and human papillomavirus vaccinations [26] and thus must be taken into consideration by the Chinese authorities.

## Conclusion

In summary, the study identified that the two surveyed populations differ in maternal age, BMI, education, immunization status, and common pregnancy affecting disease pattern, as well as the additional demands to the existing preconception service. The results suggest the following. First, the existing preconception health care service is effective on correcting unhealthy life styles. Second, more scientific education and training are required for both reproductive-age women and doctors, to enrich the knowledge of the public and also to ensure the quality of the service provided by the medical professionals. Third, regional government should design a local version of the preconception health care service guideline, which could be implemented along with the national one to best fit the physical, psychological, and environmental requirements of the local population. Fourth, distribution of general medical services is unbalanced, with an over-concentration of centers with top service standards in developed areas. The standard of medical services in under-developed areas demands urgent improvement.

## Abbreviations

BMI: Body mass index
STD: Sexually transmitted disease
HBV: Hepatitis B virus
MMR: Measles, mumps, and rubella
ETS: Environmental tobacco smoke.
